# Levels and Change in Galectin‐3 and Association With Cardiovascular Events: The ARIC Study

**DOI:** 10.1161/JAHA.119.015405

**Published:** 2020-06-23

**Authors:** David Aguilar, Caroline Sun, Ron C. Hoogeveen, Vijay Nambi, Elizabeth Selvin, Kunihiro Matsushita, Anum Saeed, John W. McEvoy, Amil M. Shah, Scott D. Solomon, Eric Boerwinkle, Christie M. Ballantyne

**Affiliations:** ^1^ Department of Epidemiology, Human Genetics, and Environmental Sciences School of Public Health University of Texas Health Science Center at Houston TX; ^2^ Division of Cardiovascular Medicine Department of Medicine Brigham and Women’s Hospital Boston MA; ^3^ Section of Cardiology Department of Medicine Baylor College of Medicine Houston TX; ^4^ Section of Cardiovascular Research Department of Medicine Baylor College of Medicine Houston TX; ^5^ Center for Cardiometabolic Disease Prevention Department of Medicine Baylor College of Medicine Houston TX; ^6^ Department of Medicine Michael E. DeBakey Veterans Affairs Medical Center Houston TX; ^7^ Department of Epidemiology Johns Hopkins Bloomberg School of Public Health Baltimore MD; ^8^ Department of Medicine Ciccarone Center for the Prevention of Heart Disease Johns Hopkins School of Medicine Baltimore MD; ^9^ National University of Ireland and National Institute for Prevention and Cardiovascular Health Galway Ireland; ^10^ Heart and Vascular Institute University of Pittsburgh Medical Center Pittsburgh PA

**Keywords:** adverse cardiovascular events, galectin‐3, heart failure, prognosis, risk, Biomarkers, Fibrosis, Risk Factors, Cardiovascular Disease, Heart Failure

## Abstract

**Background:**

Circulating galectin‐3 levels provide prognostic information in patients with established heart failure (HF), but the associations between galectin‐3 levels and other incident cardiovascular events in asymptomatic individuals at midlife and when remeasured ≈15 years later are largely uncharacterized.

**Methods and Results:**

Using multivariable Cox proportional hazards models, we identified associations between plasma galectin‐3 levels (hazard ratio [HR] per 1 SD increase in natural log galectin‐3) and incident coronary heart disease, ischemic stroke, HF hospitalization, and total mortality in ARIC (Atherosclerosis Risk in Communities) participants free of cardiovascular disease at ARIC visit 4 (1996–1998; n=9247) and at ARIC visit 5 (2011–2013; n=4829). Higher galectin‐3 level at visit 4 (median age 62) was independently associated with incident coronary heart disease (adjusted HR, 1.30; 95% CI, 1.06–1.60), ischemic stroke (HR, 1.42; 95% CI, 1.01–2.00), HF (HR, 1.44; 95% CI, 1.17–1.76), and mortality (HR, 1.56; 95% CI, 1.35–1.80). At visit 5 (median age, 74), higher galectin‐3 level was associated with incident HF (HR, 1.93; 95% CI, 1.15–3.24) and total mortality (HR, 1.70; 95% CI, 1.15–2.52), but not coronary heart disease or stoke. Individuals with the greatest increase in galectin‐3 levels from visit 4 to visit 5 were also at increased risk of incident HF and total mortality.

**Conclusions:**

In a large, biracial community‐based cohort, galectin‐3 measured at midlife and older age was associated with increased risk of cardiovascular events. An increase in galectin‐3 levels over this period was also associated with increased risk.

**Nonstandard Abbreviations and Acronyms**
ARIC: Atherosclerosis Risk in Communities
CHD: coronary heart disease
CVD: cardiovascular disease
eGFR: estimated glomerular filtration rate
HF: heart failure
HR: hazard ratio
hs‐TnI: high‐sensitivity cardiac troponin I
hs‐TnT: high‐sensitivity cardiac troponin T
*ICD‐9*: * International Classification of Diseases, Ninth Revision*
NT‐proBNP: N‐terminal pro‐B‐type natriuretic peptide

**Clinical Perspective**

**What Is New?**

- When measured at midlife, elevated plasma levels of galectin‐3, a biomarker linked to inflammation and fibrosis, are associated not only with incident heart failure, but also with incident coronary heart disease, ischemic stroke, and total mortality in a community‐based cohort.
- Several factors, including cardiometabolic risk factors such as hypertension, diabetes mellitus, body mass index, and systolic blood pressure, are associated with increasing levels of galectin‐3 over ≈15 years.
- Those individuals with the highest increase in galectin‐3 over this time period are also at increased risk for incident heart failure and total mortality.

**What Are the Clinical Implications?**

- Our findings support the hypothesis that galectin‐3 may be important in the development of atherosclerosis and heart failure and provide rationale for future studies targeting fibrosis in at‐risk populations.
- We also identify modifiable risk factors associated with increased galectin‐3 levels and the potential processes of increased inflammation and fibrosis.

Galectin‐3, a beta‐galactoside–binding lectin expressed in inflammatory cells, epithelial cells, fibroblasts, and other cell types, has important regulatory roles in adhesion, inflammation, immunity, and fibrosis.^1^ Galectin‐3 functions as a paracrine signal secreted in the bloodstream and extracellular matrix and leads to macrophage and fibroblast proliferation and fibrosis. Galectin‐3 is implicated in numerous disease pathways and may play a significant role in cardiovascular disease (CVD), including heart failure (HF).^2^, ^3^, ^4^, ^5^

Epidemiologic studies have demonstrated associations of plasma galectin‐3 levels with cardiovascular events in individuals with established HF, and recent studies extend the association of galectin‐3 with incident HF and mortality to the general population.^6^, ^7^, ^8^, ^9^, ^10^, ^11^ However, these community‐based studies were limited by relatively small, homogeneous populations and may not have been adequately powered to assess associations between galectin‐3 and other manifestations of CVD, such as coronary heart disease (CHD) and ischemic stroke. Further, the association between galectin‐3 and CVD in blacks, who have greater HF rates in the United States,^12^ is not known. Additionally, most studies in the general population measured galectin‐3 at a single time point; the drivers and prognostic implications of temporal changes in galectin‐3 are not well studied.

Therefore, we sought to establish associations between galectin‐3 and incident CHD, ischemic stroke, HF, and mortality in individuals without CVD at midlife and when remeasured ≈15 years later in the ARIC (Atherosclerosis Risk in Communities) study. We also sought to determine baseline characteristics associated with galectin‐3 increase during this period and whether temporally increased galectin‐3 is associated with cardiovascular events.

## Methods

For more information on the ARIC study, see the ARIC website (https://www2.cscc.unc.edu/aric). Anonymized data are available on dbGAP (https://www.ncbi.nlm.nih.gov/gap). For further inquiries on data or analyses, contact Dr Ballantyne (cmb@bcm.edu).

### Study Population

**Figure 1:**
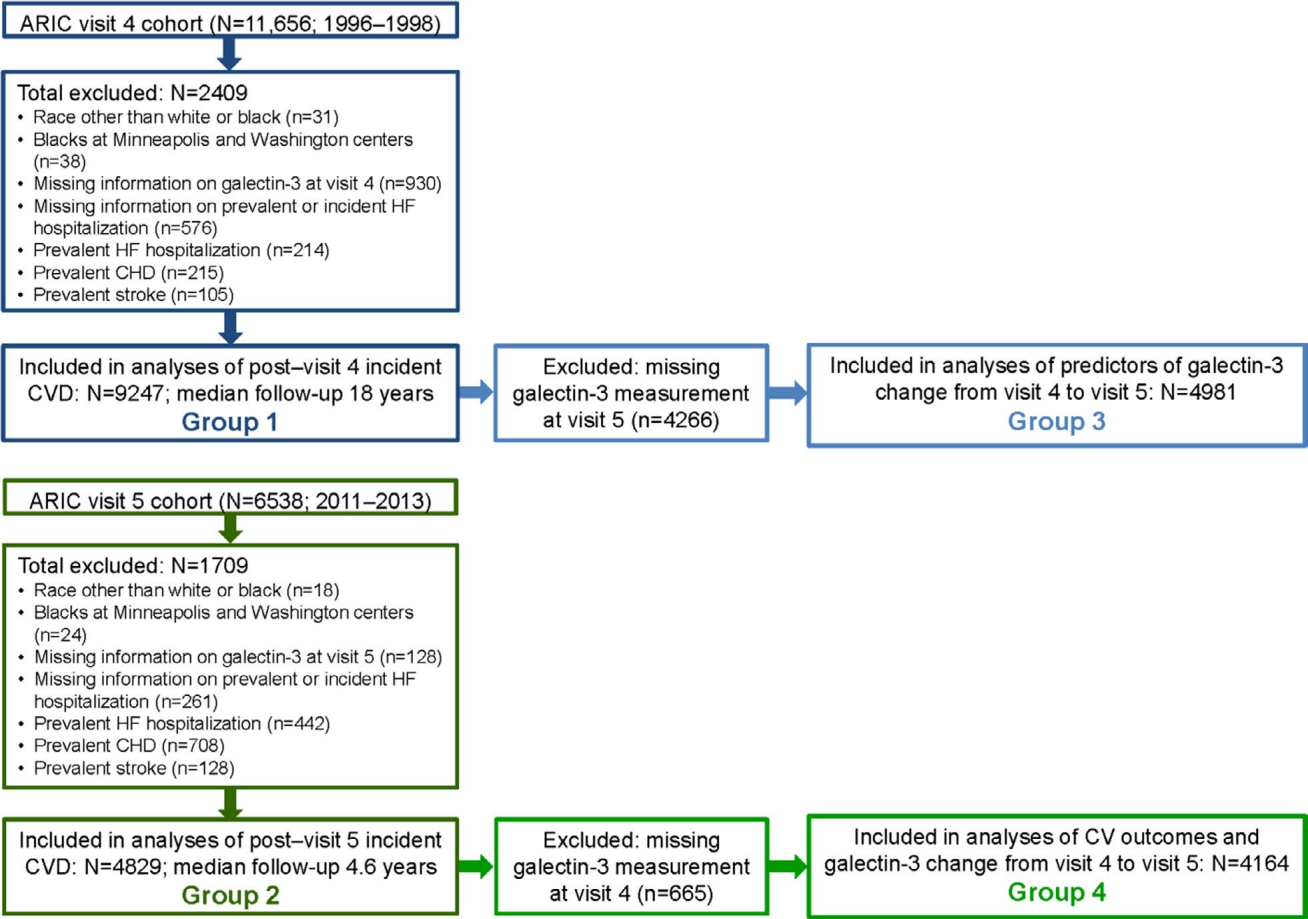
Flow diagram of study and analyses. ARIC indicates Atherosclerosis Risk in Communities; CHD, coronary heart disease; CV, cardiovascular; CVD, cardiovascular disease and HF, heart failure.

The ARIC study is an ongoing, prospective, observational cohort study that enrolled 15 792 middle‐aged adults from 4 US communities in 1987–1989.^13^ The study protocol was approved by the institutional review boards of each center, and all participants provided written informed consent. Of the 11 656 participants in visit 4 (1996–1998), we excluded those with prevalent CVD (n=834), missing information on prevalent or incident HF (n=576), missing data for galectin‐3 or other covariates in our multivariable models (n=930), and neither white nor black race (n=31), and black race in Minneapolis or Washington County (n=38), because of small numbers, resulting in 9247 individuals included in our analysis of visit 4 galectin‐3 levels and incident cardiovascular events (group 1; Figure 1). To determine baseline characteristics associated with change in galectin‐3 between visits 4 and 5 (median 15 years apart), we identified 4981 of these individuals who had galectin‐3 remeasured at visit 5 for additional analyses (group 3).

Galectin‐3 was measured in 6368 individuals at visit 5 (2011–2013). After the exclusions as for visit 4, 4829 individuals free of prevalent CVD at visit 5 were included in our analysis to determine the prognostic value of galectin‐3 at visit 5 (older age) for subsequent cardiovascular events (group 2). Finally, we assessed the prognostic value of galectin‐3 change from visit 4 to visit 5 in 4164 of these individuals who also had galectin‐3 measured at visit 4 (group 4).

### Measurement of Biomarkers

Galectin‐3 was measured using a chemiluminescent microparticle immunoassay on an Architect *i* 2000sr instrument (Abbott, Abbott Park, IL) in EDTA‐plasma samples collected at visits 4 and 5 and stored at –70°C before measurement (July 2015–February 2016 for visit 4; March–December 2017 for visit 5). The assay's limit of detection is 1.1 ng/mL; limit of quantitation is 4.0 ng/mL. Interassay coefficients of variation were 5.2%, 3.3%, and 2.3% at mean galectin‐3 levels of 8.8, 19.2, and 72.0 ng/mL, respectively. The reliability coefficient was *r*=0.92; coefficient of variation was 7.5% on the basis of 402 blinded quality‐control samples.

All covariates were assessed at visit 4 (baseline) and repeated at visit 5. Estimated glomerular filtration rate (eGFR) was estimated using the creatinine‐based Chronic Kidney Disease Epidemiology Collaboration equation.^14^ NT‐proBNP (N‐terminal pro‐B‐type natriuretic peptide) and high‐sensitivity cardiac troponin T (hs‐TnT) were measured on an automated Cobas e411 analyzer (Roche Diagnostics, Indianapolis, IN).^15^ High‐sensitivity cardiac troponin I (hs‐TnI) was measured on automated chemistry analyzer (Architect *i* 2000sr; Abbott).^16^ Hs‐CRP (High‐sensitivity C‐reactive protein) was measured with an immunonephelometric assay on a BNIII analyzer (Siemens Healthcare Diagnostics, Deerfield, IL).^17^

### Outcomes

Outcomes assessed were incident CHD (fatal CHD, definite or probable myocardial infarction, or coronary revascularization), ischemic stroke, HF hospitalization, and total mortality.^18^, ^19^, ^20^ The methods for ascertainment of cardiovascular events in ARIC have been previously published.^18^, ^19^, ^20^ In ARIC, hospitalizations were reportedly annually by participants or their proxy and identified by surveillance of hospitals in each ARIC community. Trained personnel abstracted records for potential cardiovascular events, and vital records were examined for all deaths. Incident CHD was defined as fatal CHD, definite or probable myocardial infarction, and coronary revascularization and was based on committee adjudication of abstracted records. Possible strokes were abstracted from hospital records (*International Classification of Diseases, Ninth Revision* [*ICD‐9*], codes 430–437). Using hospital records and neuroimaging reports, each eligible stroke case was classified by a computer algorithm and by a physician reviewer.^18^ Hospitalization for HF was identified by HF discharge codes (*ICD‐9,* code 428). After 2004, HF events were additionally adjudicated by an expert panel. Participants were administratively censored for events on December 31, 2016.

### Statistical Analysis

Baseline characteristics for the study population were tabulated by galectin‐3 quartiles. Continuous variables are presented as means±SDs or medians (25th, 75th percentiles); categorical variables are presented as percentages. *P* values for linear trends were calculated by using trend test across ordered groups. Spearman's rank correlation coefficients were used to determine the relationship between galectin‐3 and other cardiac biomarkers.

Because of nonnormality, galectin‐3 was natural log‐transformed in analyses to determine associations between incident cardiovascular events and galectin‐3 levels at visits 4 and 5. Cox proportional hazards regression models were used to estimate the hazard ratios (HRs) and 95% CIs for outcomes. The proportional hazards assumptions of the Cox models were verified by time‐varying covariates and likelihood ratio tests. For CHD, ischemic stroke, and total mortality, model 1 included age, sex, race, total cholesterol, high‐density lipoprotein cholesterol, systolic blood pressure, antihypertensive medication use, current smoking, and diabetes mellitus status (Pooled Cohort Equation variables).^21^ For HF hospitalization, model 1 included age, sex, race, systolic blood pressure, antihypertensive medication use, current smoking status, diabetes mellitus status, body mass index, and heart rate (ARIC HF Risk Calculator variables^22^). Model 2 was model 1 variables plus eGFR; model 3 was model 1 variables plus hs‐TnT and NT‐proBNP, biomarkers that have been shown to improve cardiovascular risk prediction in prior ARIC studies.^15^, ^23^, ^24^ Interactions between galectin‐3 and sex or race were tested by adding the interaction term into each Cox model (model 1).

Additionally, we assessed the relationship between galectin‐3 quartiles at each visit and adverse cardiovascular outcomes. Incidence rates for each outcome were calculated as the number of cardiovascular events per 1000 person‐years. We used the Kaplan–Meier method and log‐rank test to evaluate cumulative incidence of each cardiovascular event across galectin‐3 quartiles. For each cardiovascular outcome, we used the Cox proportional hazard regression models described above to adjust for other cardiovascular risk factors. We tested linear trends in HRs across galectin‐3 quartiles by using Wald chi‐squared tests.

Restricted cubic splines were used to depict nonlinear associations between galectin‐3 and each outcome. The median galectin‐3 value (14.1 ng/mL) was used as reference in a Cox proportional hazard model adjusted for variables described above for model 1. The knots were placed at the 5th, 27.5th, 50th, 72.5th, and 95th percentiles.

To analyze incremental value of visit 4 galectin‐3 in risk prediction, area under the receiver operating characteristic curve, continuous net reclassification improvement (NRI), and integrated discrimination improvement were calculated comparing model 1 (traditional risk factors) with and without galectin‐3.^25^

To determine characteristics at visit 4 associated with temporal increase in galectin‐3, we categorized individuals into quartiles of absolute change in galectin‐3 from visit 4 to visit 5 (group 3). We used multivariable linear regression to determine baseline (visit 4) variables associated with absolute change in galectin‐3 from visit 4 to visit 5. From a list of candidate variables, we used backward elimination to create a multivariable linear regression of visit 4 variables associated with absolute change from visit 4 to visit 5. Finally, we determined the prognostic significance of absolute change in galectin‐3 between visits 4 and 5 in individuals free of incident CVD at visit 5 (group 4) by using multivariable models as described above. SAS version 9.4 (SAS Institute Inc., Cary, NC) and Stata version 12 (StataCorp, College Station, TX) were used for the analyses. All tests were 2‐tailed with *P*<0.05 considered statistically significant.

Drs Aguilar and Ballantyne had full access to all the data in the study and accept responsibility for its integrity and the data analysis.

## Results

Baseline (visit 4) characteristics are shown by galectin‐3 quartiles (Table 1); all galectin‐3 values were above the limit of quantitation (4.0 ng/mL). The median age at visit 4 was 62 years. Individuals with higher galectin‐3 were older, more often female, and more often black and had higher body mass index and higher prevalence of diabetes mellitus, hypertension, and left ventricular hypertrophy. Systolic blood pressure, total cholesterol, triglycerides, and high‐density lipoprotein cholesterol increased across galectin‐3 quartiles, and eGFR decreased across increasing quartiles. hs‐TnT, hs‐TnI, NT‐proBNP, and hs‐CRP levels increased with increasing galectin‐3. Galectin‐3 was correlated with hs‐CRP (*r*_s_=0.24, *P*<0.0001) and NT‐proBNP (*r*_s_=0.16, *P*<0.0001) and weakly correlated with hs‐TnT (*r*_s_=0.05, *P*<0.0001) and hs‐TnI (*r*_s_=0.05, *P*<0.0001). Galectin‐3 was also negatively correlated with eGFR (*r*_s_=–0.21, *P*<0.0001). Correlations with other cardiovascular risk factors are shown in Table S1.

**Table 1:** Baseline Characteristics by Galectin‐3 Quartiles at Visit 4 (n=9247)

Risk Factors	Galectin‐3 Quartiles (ng/mL)				*P* Trend
	First Quartile (4.4–11.9) n=2344	Second Quartile (12.0–14.1) n=2326	Third Quartile (14.2–16.7) n=2301	Fourth Quartile (16.8–114) n=2276	
Age, y	61.5±5.4	62.2±5.6	62.8±5.6	64.0±5.7	<0.001
Female, %	40.3	53.1	64.3	74.8	<0.001
Black, %	17.0	20.2	22.6	27.7	<0.001
SBP, mm Hg	125±18	126±18	127±19	129±20	<0.001
Pulse pressure, mm Hg	54±15	55±15	56±15	59±17	<0.001
Heart rate, beats/min	65±9	66±9	66±10	67±10	<0.001
Hypertension, %	36.0	41.4	44.4	56.9	<0.001
Current smoking, %	14.5	15.4	13.2	14.7	0.60
Diabetes mellitus, %	13.7	12.5	15.2	18.2	<0.001
BMI, kg/m^2^	27.9±4.7	28.0±5.0	28.8±5.5	29.8±6.4	<0.001
Triglycerides, mg/dL	115 (83, 164)	117 (86, 165)	122 (90, 173)	130 (93, 186)	<0.001
Total cholesterol, mg/dL	198.0±34.5	200.8±35.3	202.9±37.0	204.9±38.3	<0.001
HDL‐C, mg/dL	49.5±16.2	50.7±16.6	51.2±16.9	51.7±16.9	<0.001
LDL‐C, mg/dL	122.0±31.4	123.4±33.0	123.4±33.4	123.3±34.6	0.29
eGFR, mL/min per 1.73 m^2^	90.5±13.0	88.8±14.0	86.9±14.5	81.0±18.6	<0.001
Chronic kidney disease (eGFR <60 mL/min per 1.73 m^2^), %	2.0	2.6	4.0	13.3	<0.001
NT‐proBNP, pg/mL	52.1 (26.0, 102.5)	61.2 (29.0, 119.6)	63.8 (33.1, 117.9)	84.8 (42.7, 152.8)	<0.001
hs‐TnT, ng/L	4 (1.5, 7)	5 (1.5, 7)	4 (1.5, 8)	5 (1.5, 9)	<0.001
hs‐TnI, ng/L	2.1 (1.5, 3.2)	2.1 (1.4, 3.3)	2.1 (1.4, 3.2)	2.4 (1.6, 3.8)	<0.001
hs‐CRP, mg/L	1.6 (0.8, 3.7)	2.0 (1.0, 4.5)	2.7 (1.2, 5.6)	3.8 (1.6, 7.5)	<0.001
LVH, %	2.4	2.9	3.0	4.2	0.004

Data are presented as mean±SD, median (25th percentile, 75th percentile), or percentage. *P* values for linear trend were calculated by trend test across ordered groups. BMI indicates body mass index; eGFR, estimated glomerular filtration rate; HDL‐C, high‐density lipoprotein cholesterol; hs‐CRP, high‐sensitivity C‐reactive protein; hs‐TnI, high‐sensitivity troponin I; hs‐TnT, high‐sensitivity troponin T; LDL‐C, low‐density lipoprotein cholesterol; LVH, left ventricular hypertrophy (Cornell ECG criteria); NT‐proBNP, N‐terminal pro‐B‐type natriuretic peptide; and SBP, systolic blood pressure.

### Midlife Galectin‐3 (Visit 4) and Cardiovascular Events at Long‐Term Follow‐Up

**Figure 2:**
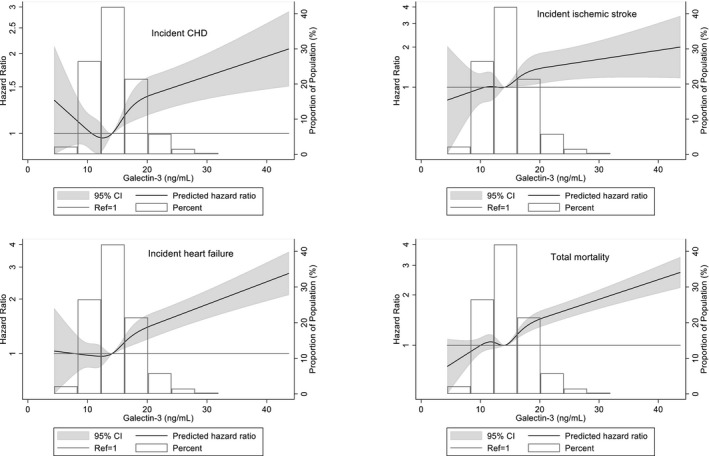
Associations of galectin‐3 with incident coronary heart disease (CHD), ischemic stroke, heart failure hospitalization, and mortality in ARIC (1996–2016). Median galectin‐3 (14.1 ng/mL) was used as reference in a Cox proportional hazard model adjusted for age, sex, race, total cholesterol, high‐density lipoprotein cholesterol, systolic blood pressure, antihypertensive medication, current smoking, diabetes mellitus status, and estimated glomerular filtration rate for CHD, ischemic stroke, and total mortality; adjusted for age, sex, race, body mass index, heart rate, systolic blood pressure, antihypertensive medication, current smoking, diabetes mellitus status, and estimated glomerular filtration rate for heart failure. High extreme values of galectin‐3 (>45 ng/mL; n=10) were excluded from the model. The knots were placed at the 5th, 27.5th, 50th, 72.5th, and 95th percentiles. ARIC indicates Atherosclerosis Risk in Communities; CHD, coronary heart disease; CVD, cardiovascular disease; and HF, heart failure.

During ≈18‐year median follow‐up after visit 4, 1570 individuals had incident CHD, 564 had incident stroke, 1599 had incident HF, and 3282 died. After adjusting for traditional cardiovascular risk factors (model 1), log galectin‐3 levels were predictive of incident CHD, ischemic stroke, HF hospitalization, and mortality (Table 2). These relationships remained significant when eGFR (model 2) and NT‐proBNP and hs‐TnT (model 3) were added. Restricted cubic spline analyses (Figure 2) demonstrated continuous relationships between visit 4 galectin‐3 and HRs (adjusted for age, sex, and race) for each outcome. HRs for each outcome increased above the median galectin‐3 level (14.1 ng/mL).

**Table 2:** Associations of Log Galectin‐3 and Incident Cardiovascular Events at Visits 4 and 5

	Hazard Ratio (95% CI)			
	Coronary Heart Disease	Ischemic Stroke	Heart Failure Hospitalization	Death
ARIC visit 4				
Model 1	1.67 (1.37–2.04), *P*<0.001	1.91 (1.37–2.66), *P*<0.001	2.23 (1.82–2.73), *P*<0.001	1.94 (1.69–2.23), *P*<0.001
Model 2	1.56 (1.27–1.92), *P*<0.001	1.74 (1.23–2.46), *P*=0.002	1.98 (1.60–2.43), *P*<0.001	1.87 (1.62–2.16), *P*<0.001
Model 3	1.30 (1.06–1.60), *P*=0.01	1.42 (1.01–2.00), *P*=0.044	1.44 (1.17–1.76), *P*<0.001	1.56 (1.35–1.80), *P*<0.001
ARIC visit 5				
Model 1	1.96 (1.07–3.60), *P*=0.03	1.32 (0.60–2.90), *P*=0.48	3.90 (2.47–6.17), *P*<0.001	2.65 (1.86–3.77), *P*<0.001
Model 2	1.68 (0.86–3.26), *P*=0.13	1.31 (0.55–3.11), *P*=0.54	3.31 (1.98–5.54), *P*<0.001	2.39 (1.62–3.53), *P*<0.001
Model 3	1.33 (0.68–2.60), *P*=0.4	1.02 (0.43–2.43), *P*=0.97	1.93 (1.15–3.24), *P*=0.01	1.70 (1.15–2.52), *P*=0.008

Hazard ratios (95% CIs) denote hazard associated with a 1‐SD increase in log galectin‐3 levels (SD of log galectin‐3=log galectin‐3/0.265). Model 1 was adjusted by age, sex, race, total cholesterol, high‐density lipoprotein cholesterol, systolic blood pressure, antihypertensive medication, current smoking, and diabetes mellitus status except for heart failure, for which model 1 included age, sex, race, systolic blood pressure, antihypertensive medication, current smoking, diabetes mellitus status, body mass index, and heart rate. Model 2 was model 1 plus estimated glomerular filtration rate. Model 3 was model 2 plus log N‐terminal pro‐B‐type natriuretic peptide and log high‐sensitivity cardiac troponin T. ARIC indicates Atherosclerosis Risk in Communities.

HRs for cardiovascular events and Kaplan–Meier survival curves by galectin‐3 quartiles are shown in Table S2 and Figure S1, respectively. For each cardiovascular outcome, individuals in the highest quartile of galectin‐3 had increased risk compared with those in the lowest quartile in adjusted models.

### Analyses by Sex and Race at Visit 4

Median (25th, 75th percentiles) galectin‐3 levels were higher in women (14.9 [12.7, 14.6] ng/L) than men (13.0 [11.1, 15.3] ng/L; *P*=0.0001). No statistical interaction was found between log galectin‐3 and sex for any outcome (*P*_interaction_ >0.10 for all).

Median galectin‐3 levels were higher in blacks (14.8 [12.5, 17.5] ng/L) than whites (13.9 [11.8, 16.5] ng/L; *P*=0.0001). No statistical interaction was found between log galectin‐3 and race for any outcome (*P*_interaction_ >0.09 for all). Similarly, there was no statistical interaction for race and incident stroke, HF hospitalization, or mortality in analyses by galectin‐3 quartiles (*P*_interaction_ >0.16 for all), and only a borderline interaction by race for incident CHD in quartile analyses (*P*_interaction_=0.02).

### Risk Reclassification and Discrimination With Galectin‐3 at Visit 4

The addition of galectin‐3 to traditional risk prediction models (Pooled Cohort Equation or ARIC HF Risk Calculator) improved area under the receiver operating characteristic curve, net reclassification improvement, and integrated discrimination improvement for all outcomes (Table 3). Improvements in area under the receiver operating characteristic curve and net reclassification improvement were modest for incident CHD and ischemic stroke; the largest difference was for incident HF hospitalization.

**Table 3:** Model Comparisons for Risk Prediction of Cardiovascular Events at ARIC Visit 4

	AUC Basic^a^ (95% CI)	AUC Extension^a^ (95% CI)	AUC Difference^b^ (95% CI)	Continuous NRI^b^ (95% CI)	IDI^b^ (95% CI)
Incident CHD	0.7102 (0.6991–0.7237)	0.7139 (0.7027–0.7287)	0.0038 (0.0014–0.0071)	0.1385 (0.0696–0.2055)	0.0036 (0.0015–0.0069)
Incident ischemic stroke	0.7162 (0.6986–0.7397)	0.7207 (0.7029–0.7433)	0.0045 (0.0007–0.0098)	0.1487 (0.0049–0.2572)	0.0022 (0.0005–0.0053)
Incident HF	0.7455 (0.7356–0.7592)	0.7523 (0.7415–0.7671)	0.0068 (0.0034–0.0113)	0.2660 (0.1980–0.3563)	0.0082 (0.0043–0.0137)
Death	0.7339 (0.7242–0.7432)	0.7392 (0.7296–0.7499)	0.0053 (0.0031–0.0082)	0.1686 (0.0888–0.2237)	0.0068 (0.0039–0.0101)

ARIC indicates Atherosclerosis Risk in Communities; AUC, area under the receiver operating characteristic curve; CHD, coronary heart disease; HF, heart failure; IDI, integrated discrimination index; and NRI, net reclassification improvement.

a The basic models for CHD, stroke, and death (Pooled Cohort Equation model) were adjusted by age, sex, race, total cholesterol, high‐density lipoprotein cholesterol, systolic blood pressure, antihypertensive medication use, current smoking, and diabetes mellitus status; the basic model for incident HF (ARIC HF model) was adjusted by age, sex, race, heart rate, body mass index, systolic blood pressure, antihypertensive medication use, current smoking, and diabetes mellitus status. The extension models included log galectin‐3.

b Extension model vs basic model.

### Visit 5 Galectin‐3 and Cardiovascular Events

The median age of the cohort at visit 5 (group 2) was 74 years. Associations between increasing galectin‐3 quartiles and visit 5 characteristics (Table S3) were similar to those at visit 4. At visit 5, galectin‐3 was correlated with hs‐CRP (*r*_s_=0.15, *P*<0.0001), NT‐proBNP (*r*_s_=0.21, *P*<0.0001), hs‐TnT (*r*_s_=0.19, *P*<0.0001), and hs‐TnI (*r*_s_=0.19, *P*<0.0001). Galectin‐3 at visit 4 was correlated with galectin‐3 at visit 5 (*r*_s_=0.71, *P*<0.0001).

During 4.6‐year median follow‐up, 126 individuals had incident CHD, 89 had incident stroke, 217 had incident HF, and 384 died. In this older cohort, log galectin‐3 levels predicted incident HF hospitalization and mortality in the 3 adjusted models (Table 2). In quartile analyses, individuals in the highest galectin‐3 quartile had increased risk for HF hospitalization and death compared with those in the lowest quartile when adjusted for traditional cardiovascular risk factors (model 1) and eGFR (model 2), but it was no longer significant after adjustment for NT‐proBNP and hs‐TnT (model 3) (Table S4). Galectin‐3 levels at visit 5, when analyzed as log galectin (Table 2), were not associated with incident CHD after adjustment for models that included traditional risk factors and eGFR (model 2) or in quartile analyses (Table S4). Galectin‐3 at visit 5 was also not associated with incident ischemic stroke (Table 2 and Table S4).

### Change in Galectin‐3 Levels From Midlife to Older Age

Among ARIC participants with galectin‐3 measured at visit 4 and remeasured ≈15 years later at visit 5 (group 3), galectin‐3 increased by (median [25th, 75th percentiles]) 2.7 (1.0, 5.0) ng/L. Table S5 presents visit 4 characteristics by quartiles of absolute change in galectin‐3 level from visit 4 to visit 5. In multivariable models, the absolute change in galectin‐3 was positively associated with baseline log galectin‐3, age, black race, male sex, fasting glucose, diabetes mellitus, body mass index, current smoking status, systolic blood pressure, pulse pressure, antihypertensive medication, log NT‐proBNP, and log hs‐TnI (Table 4).

**Table 4:** Multivariable Regression Models of Risk Factors at Visit 4 Associated With Absolute Change in Galectin‐3 Levels From Visit 4 to Visit 5

Variable	Coefficient (95% CI)	*P* Value
Log galectin‐3 (per log unit increase)	0.94 (0.38–1.49)	0.001
Age, y	0.06 (0.03–0.09)	<0.001
Female	−0.55 (−0.87 to −0.22)	0.001
Black	0.44 (0.09–0.80)	0.01
Current smoking	0.75 (0.33–1.17)	<0.001
Systolic blood pressure, mm Hg	0.02 (0.002–0.03)	0.03
Pulse pressure, mm Hg	0.02 (0.003–0.04)	0.02
Antihypertensive medication	0.32 (0.03–0.61)	0.03
Body mass index, kg/m^2^	0.08 (0.05–0.11)	<0.001
Diabetes mellitus	0.81 (0.19–1.42)	0.01
Fasting glucose, mg/dL	0.03 (0.02–0.03)	<0.001
Log NT‐proBNP (per log unit increase)	0.41 (0.27–0.55)	<0.001
Log hs‐TnI (per log unit increase)	0.23 (0.04–0.42)	0.02
Log hs‐TnT (per log unit increase)	0.19 (−0.002 to 0.38)	0.05

hs‐TNI indicates high‐sensitivity cardiac troponin I; hs‐TNT, high‐sensitivity cardiac troponin T; and NT‐proBNP, N‐terminal pro‐B‐type natriuretic peptide.

Over the median follow‐up of 4.6 years after visit 5, individuals without evidence of CVD or HF at visit 5 (group 4) who were in the highest quartile of absolute galectin‐3 change between visits 4 and 5 had increased hazard of incident HF after adjustment for traditional HF risk factors (model 1: HR, 2.17; 95% CI, 1.42–3.32; *P*<0.0001) when compared with those in the lowest quartile (Table 5). This relationship persisted when eGFR was added (model 2: HR, 1.74; 95% CI, 1.10–2.76; *P*=0.01,) but this was no longer statistically significant when NT‐proBNP and hs‐TnT were added (model 3: HR, 1.20; 95% CI, 0.74–1.93; *P*=0.51). Similarly, individuals in the highest quartile had increased risk of death that persisted after adjustment for traditional risk factors (model 1: HR, 1.68; 95% CI, 1.23–2.29; *P*=0.0004) and eGFR (model 2: HR, 1.55; 95% CI, 1.11–2.17; *P*=0.009), but the HR was no longer statistically significant when adjusted for NT‐proBNP and hs‐TnT (model 3: HR, 1.24; 95% CI, 0.88–1.75; *P*=0.29). Results were similar when galectin‐3 change was modeled as percentage change from visit 4 to visit 5 (data not shown).

**Table 5:** Cardiovascular Outcomes by Quartiles in Change of Galectin‐3 From ARIC Visit 4 to Visit 5 (Group 4; N=4164)

	Quartiles of Absolute Change in Galectin‐3 From Visit 4 to Visit (ng/mL)				*P* Trend
	First Quartile (−32.4 to 1.0, n=1083)	Second Quartile (1.00–2.7, n=1005)	Third Quartile (2.8–4.99, n=1036)	Fourth Quartile (5.0–61.5, n=1040)	
Incident CHD					
Incident rate	5.82 (4.05–8.38)	5.84 (3.97–8.57)	8.03 (5.79–11.13)	8.11 (5.82–11.29)	0.09
HR‐model 1	Ref	0.88 (0.51–1.53)	1.12 (0.67–1.89)	1.05 (0.61–1.80)	0.85
HR‐model 2	Ref	0.85 (0.49–1.48)	1.04 (0.61–1.76)	0.83 (0.46–1.49)	0.79
HR‐model 3	Ref	0.83 (0.48–1.45)	0.97 (0.58–1.65)	0.70 (0.38–1.27)	0.58
Incident ischemic stroke					
Incident rate	3.81 (2.43–5.97)	4.02 (2.53–6.38)	4.19 (2.67–6.57)	6.93 (4.85–9.91)	0.04
HR‐model 1	Ref	0.83 (0.42–1.63)	0.90 (0.47–1.74)	1.15 (0.61–2.15)	0.78
HR‐model 2	Ref	0.82 (0.41–1.61)	0.88 (0.46–1.71)	1.05 (0.53–2.08)	0.87
HR‐model 3	Ref	0.81 (0.41–1.61)	0.85 (0.44–1.64)	0.89 (0.44–1.79)	0.94
Incident HF hospitalization					
Incident rate	7.23 (5.22–10.02)	8.54 (6.21–11.73)	9.99 (7.46–13.38)	23.20 (19.03–28.28)	<0.0001
HR‐model 1	Ref	1.02 (0.62–1.67)	1.03 (0.63–1.66)	2.17 (1.42–3.32)	<0.0001
HR‐model 2	Ref	0.99 (0.60–1.62)	0.96 (0.59–1.56)	1.74 (1.10–2.76)	0.01
HR‐model 3	Ref	0.92 (0.56–1.51)	0.88 (0.55–1.44)	1.20 (0.74–1.93)	0.51
Death					
Incident rate	15.51 (12.43–19.37)	15.31 (12.09–19.38)	19.71 (16.03–24.23)	33.55 (28.54–39.44)	<0.0001
HR‐model 1	Ref	0.94 (0.66–1.32)	1.10 (0.80–1.53)	1.68 (1.23–2.29)	0.0004
HR‐model 2	Ref	0.93 (0.66–1.31)	1.08 (0.78–1.50)	1.55 (1.11–2.17)	0.009
HR‐model 3	Ref	0.90 (0.64–1.28)	1.03 (0.74–1.43)	1.24 (0.88–1.75)	0.29

Incidence rates are presented as rate/1000 person years (95% CI). Model data are presented as HR (95% CI). For CHD, stroke, and death, model 1 was adjusted by age, sex, race, total cholesterol, high‐density lipoprotein cholesterol, systolic blood pressure, antihypertensive medication, current smoking, and diabetes mellitus status; for HF, model 1 was the ARIC HF model and included age, sex, race, systolic blood pressure, antihypertensive medication, current smoking, diabetes mellitus status, body mass index, and heart rate. Model 2 was model 1 plus estimated glomerular filtration rate. Model 3 was model 2 plus log N‐terminal pro‐B‐type natriuretic peptide and log high‐sensitivity cardiac troponin T. *P* trend for linearity of HR of proportional hazard regression model is calculated based on the results of Wald chi‐squared test on linearity hypothesis of ordered galectin‐3 quartiles. ARIC indicates Atherosclerosis Risk in Communities; CHD, coronary heart disease; HF, heart failure; and HR, hazard ratio.

## Discussion

In this community‐based population of individuals initially without CVD who were followed for ≈18 years, galectin‐3 measured at midlife was significantly associated with incident CHD, ischemic stroke, HF hospitalization, and mortality after adjustment for traditional risk factors, renal function, and other cardiac biomarkers. Galectin‐3 measured at older age also provided prognostic information and was associated with incident HF hospitalization and mortality over a shorter follow‐up.

While other studies have reported associations of galectin‐3 levels with death^6^, ^7^, ^10^, ^11^ and incident HF^8^, ^9^, ^10^, ^11^ in the general population, our study expands previous work by identifying an adverse relationship between CHD and ischemic stroke. Despite the potential physiologic role of galectin‐3 in atherosclerosis,^26^ galectin‐3 was not associated with CHD risk in some previous studies,^6^, ^11^ possibly attributable to fewer events and lower statistical power in prior studies; in our study, with 1570 incident CHD events over 18 years of follow‐up, higher galectin‐3 at midlife was associated with incident CHD even after adjustment for traditional risk factors, other cardiac biomarkers, and renal function. Galectin‐3 below the 25th percentile was also associated with reduced 3‐year CHD and CVD risk in elderly BioImage participants.^27^ Furthermore, our study extends the risk associated with galectin‐3 measured at midlife to ischemic stroke. A previous study in a Finnish population demonstrated a trend toward increased stroke risk across galectin‐3 quartiles that was not significant after adjustment for traditional risk factors.^11^ In ARIC, the association between galectin‐3 and ischemic stroke remained after adjustments for traditional risk factors, eGFR, NT‐proBNP, and hs‐TnT.

The mechanisms through which galectin‐3 may increase risk of atherosclerotic events are not completely understood. Galectin‐3 has been shown to be involved in experimental animal models of atherosclerosis,^26^ potentially mediated by proinflammatory effects of galectin‐3.^26^, ^28^ Galectin‐3 has also been associated with fatal cardiovascular events in patients with established CHD, suggesting a potential role in plaque instability/rupture.^29^ In studies further implicating galectin‐3 in atherosclerosis, galectin‐3 inhibition in experimental mice models of atherosclerosis reduced atherosclerotic plaque progression and inflammation.^30^

Consistent with prior studies, our study also demonstrated that the risk associated with galectin‐3 appeared higher for HF than other outcomes.^8^, ^9^, ^10^, ^11^ In the Framingham Offspring Study, log galectin‐3 was associated with incident HF in models adjusted for traditional risk factors and B‐type natriuretic peptide, but the association was attenuated and only marginally significant after adjustment for eGFR.^9^ With longer follow‐up and more HF hospitalizations in ARIC, we extend previous observations by demonstrating that the association between midlife galectin‐3 and incident HF remained statistically significant after adjustment for eGFR and other biomarkers of HF risk (NT‐proBNP and hs‐TnT) and that galectin‐3 was associated with incident HF in both white and black individuals.

Additionally, we demonstrated that higher galectin‐3 levels, when analyzed as a continuous variable, were associated with incident HF and total mortality in more short‐term follow‐up (median follow‐up of 4.6 years) when measured in older individuals (median age, 74 years) without prevalent CVD. The adverse relationships between log galectin‐3 and incident HF and total mortality persisted after adjustments for traditional risk factors, renal function, and other cardiac biomarkers. Unlike galectin‐3 measurement at midlife, we did not find a statistically significant association between higher galectin‐3 levels and CHD or stroke in this group of older individuals. This lack of association for the outcomes of CHD or stroke may be related to lower statistical power because of the smaller sample size, shorter duration of follow‐up, and lower number of CHD events at ARIC visit 5 compared with visit 4.

Using multivariable models, we identified several factors associated with greater increases in galectin‐3 levels over ≈15 years: age, male sex, black race, current smoking, blood pressure (systolic blood pressure, pulse pressure, antihypertensive medications), metabolic risk factors (fasting glucose, diabetes mellitus, body mass index), and cardiac biomarkers (baseline galectin‐3, NT‐proBNP, hs‐TnI). In a Dutch community‐based cohort, increased systolic blood pressure and urinary albumin excretion were associated with increased galectin‐3 levels over time,^31^ and the Framingham Offspring Study demonstrated that older age, female sex, systolic blood pressure, diabetes mellitus, and body mass index were associated with longitudinal increase in galectin‐3.^32^ Our study expands this previous work and implicates these potentially modifiable risk factors and pathophysiologic pathways that may contribute to elevated galectin‐3 levels years later in a diverse, community‐based cohort. Importantly, increased galectin‐3 over time was associated with incident HF and total mortality over a relatively short period, even after accounting for traditional cardiovascular risk factors. These findings are similar to the Framingham study, in which longitudinal increase in galectin‐3 was associated with incident HF and total mortality, although, unlike our study, the Framingham study included individuals with prevalent CVD.^32^ While serial galectin‐3 measurements have been shown to provide prognostic information in patients with existing HF,^33^ studies in the general population are limited.^32^, ^34^

A strength of our study was the biracial cohort of the ARIC Study. Previous studies in the general population have been limited by small numbers of black participants.^6^, ^7^, ^8^, ^9^, ^11^ A previous substudy in 1809 ARIC participants demonstrated a potential interaction between race and galectin‐3 levels for a composite of incident HF or death; white, but not black, participants had increased risk of incident HF or death.^35^ In our study, which included more black participants and outcomes, galectin‐3 was associated with both incident HF and mortality in both black and white participants.

While higher galectin‐3 was associated with incident cardiovascular events, the improvement in area under the receiver operating characteristic curve for each outcome, albeit statistically significant, was modest. Net reclassification improvement improved with the addition of galectin‐3, most significantly for HF. While these findings suggest that adding galectin‐3 to current risk prediction models may not be useful for predicting CHD, they support the hypothesis that galectin‐3 and fibrosis may be important in the development of atherosclerotic disease and HF in the general population. Indeed, pharmacologic inhibition of galectin‐3 has been shown to prevent cardiac inflammation and fibrosis and prevent left ventricular dysfunction in experimental models, suggesting a direct association between galectin‐3 and cardiac dysfunction.^4^, ^5^, ^36^, ^37^ Our findings further support the development of therapeutic strategies potentially directly targeting these pathways of fibrosis and inflammation to prevent atherosclerotic disease and heart failure. Additionally, elevated galectin‐3 levels could be used as a marker of individuals at increased risk who could then be treated with specific therapies targeting these pathways. Such a biomarker approach to identify high‐risk individuals who may benefit from anti‐inflammatory therapy to reduce adverse cardiovascular events was used in the CANTOS (Canakinumab Antiinflammatory Thrombosis Outcome Study).^38^

Strengths of this study include the large sample size, large number of clinical events, biracial population, and 2 measurements of galectin‐3 over a 15‐year period. Despite these strengths, several limitations should be acknowledged. We used stored samples for galectin‐3 measurements at 2 time points separated by ≈15 years. It is possible that degradation may have occurred, although a prior study has demonstrated stability of galectin‐3 in stored samples, albeit at a shorter interval.^39^ Another possible limitation of the study at visit 5 is potential bias resulting from loss to follow‐up over this prolonged period. Finally, despite adjusting for multiple confounders, residual confounding may be present.

In conclusion, plasma galectin‐3 levels were independently associated with subsequent cardiovascular risk in a community‐based population. Our findings support the hypothesis that galectin‐3 and fibrosis may be important in the development of atherosclerotic disease and HF. We also identified modifiable risk factors associated with increased galectin‐3 levels and the potential process of increased fibrosis and inflammation. Finally, longitudinal increase in galectin‐3 was associated with HF and total mortality in this biracial population. Future studies examining pathways of fibrosis may lead to new targets for prevention of atherosclerotic disease and HF.

## Sources of Funding

This work was supported by the National Institutes of Health (contract numbers HHSN268201100005C, HHSN268201100006C, HHSN268201100007C, HHSN268201100008C, HHSN268201100009C, HHSN268201100010C, HHSN268201100011C, and HHSN268201100012C and grant numbers K24DK106414 to Dr Selvin; R01DK089174 to Dr Selvin; R01HL134320 to Drs Selvin, Matsushita, and Ballantyne; K08HL116792 to Dr Shah, R01HL135008 to Dr Shah, and R01HL143224 to Dr Shah); American Heart Association (grant number 17MCPRP33400031 to Dr McEvoy); and Brigham and Women's Heart and Vascular Center (Watkins Discovery Award to Dr McEvoy). Biomarker measurements were supported by Abbott (galectin‐3) and Roche (NT‐proBNP and hs‐TnT) through grants to Baylor College of Medicine for the cost of assays.

## Disclosures

Dr Hoogeveen received a research grant from Denka Seiken (significant). Drs Hoogeveen, Nambi, and Ballantyne are named on provisional patent no. 61721475 entitled “Biomarkers to Improve Prediction of Heart Failure Risk” filed by Baylor College of Medicine and Roche (modest). Dr Shah reports receiving research support from Novartis and consulting fees from Bellerophon Therapeutics and Philips Ultrasound. Dr Ballantyne has received consulting fees from Abbott and Roche (modest).

## Supporting information


**Tables S1–S5 Figure S1**
