# Supplementary material for: Levels and Change in Galectin‐3 and Association With Cardiovascular Events: The ARIC Study
Source: J Am Heart Assoc. 2020 Jun 23;9(13):e015405. doi: 10.1161/JAHA.119.015405 (PMC7670497; doi:10.1161/JAHA.119.015405)
Supplement: Supplementary file 1 — Tables S1–S5 Figure S1 [file JAH3-9-e015405-s001.pdf]

SUPPLEMENTAL MATERIAL

Table S1. Spearman's correlation coefficients for ARIC visit 4 galectin-3 and cardiovascular disease risk factors.

	**Spearman's *r***	**P value**
Age (years)	0.17	<0.0001
hs-CRP (mg/L)	0.24	<0.0001
NT-proBNP (pg/mL)	0.16	<0.0001
hs-TnT (ng/L)	0.05	<0.0001
hs-TnI (ng/L)	0.05	<0.0001
Total cholesterol (mg/dL)	0.07	<0.0001
Triglycerides (mg/dL)	0.09	<0.0001
HDL-C (mg/dL)	0.05	<0.0001
LDL-C (mg/dL)	0.01	0.27
eGFR (mL/min/1.73m^2)	-0.21	<0.0001
Fasting glucose (mg/dL)	-0.01	0.19
ALT (u/L)	-0.08	<0.0001
Uric acid (mg/dL)	0.10	<0.0001
BMI (kg/ m^2)	0.11	<0.0001
Waist circumference (cm)	0.09	<0.0001
SBP (mm Hg)	0.08	<0.0001
DBP (mm Hg)	-0.04	0.0001
Pulse pressure (mm Hg)	0.12	<0.0001
Heart rate (beats per minute)	0.06	<0.0001

BMI, body mass index; DBP, diastolic blood pressure; eGFR, estimated glomerular filtration rate; HDL-C, high-density lipoprotein cholesterol; hs-CRP, high-sensitivity C-reactive protein; hs-TnI, high-sensitivity troponin I; hs-TnT, high-sensitivity troponin T; LDL-C, low-density lipoprotein cholesterol; LVH, left ventricular hypertrophy (Cornell ECG criteria); NT-proBNP, N-terminal pro–B-type natriuretic peptide; SBP, systolic blood pressure

Table S2. Cardiovascular outcomes by galectin-3 quartiles at ARIC visit 4.

	Quartiles of Galectin-3 (ng/mL)				
	1st quartile (4.4–11.9)	2nd quartile (12–14.1)	3rd quartile (14.2–16.7)	4th quartile (16.8–114)	P trend
**CHD**					
Incidence rates	10.8 (9.8-11.9)	10.2 (9.2-11.3)	10.1 (9.1-11.2)	13.8 (12.6-15.1)	0.001
Model 1	1 (reference)	1.01 (0.88-1.17)	1.05 (0.91-1.22)	1.45 (1.26-1.68)	<0.0001
Model 2	1 (reference)	1.01 (0.87-1.16)	1.04 (0.89-1.20)	1.40 (1.20-1.62)	<0.0001
Model 3	1 (reference)	0.98 (0.84-1.13)	0.99 (0.85-1.15)	1.29 (1.11-1.50)	0.0003
**Ischemic stroke**					
Incidence rates	2.9 (2.4-3.5)	3.5 (3.0-4.1)	3.5 (3.0-4.2)	5.4 (4.7-6.2)	<0.0001
Model 1	1 (reference)	1.15 (0.89-1.48)	1.10 (0.85-1.43)	1.52 (1.18-1.95)	0.004
Model 2	1 (reference)	1.13 (0.88-1.46)	1.08 (0.83-1.40)	1.44 (1.11-1.86)	0.021
Model 3	1 (reference)	1.09 (0.84-1.42)	1.06 (0.81-1.38)	1.38 (1.06-1.80)	0.054
**HF hospitalization**					
Incidence rates	7.9 (7.1-8.9)	9.2 (8.3-10.2)	10.7 (9.7-11.8)	16.7 (15.4-18.1)	<0.0001
Model 1	1 (reference)	1.11 (0.95-1.30)	1.17 (1.01-1.37)	1.56 (1.34-1.81)	<0.0001
Model 2	1 (reference)	1.10 (0.94-1.28)	1.13 (0.97-1.32)	1.45 (1.24-1.69)	<0.0001
Model 3	1 (reference)	1.03 (0.88-1.21)	1.05 (0.90-1.23)	1.27 (1.09-1.49)	0.004
**Total mortality**					
Incidence rates	17.7 (16.4-19.1)	20.0 (18.7-21.5)	20.0 (18.7-21.5)	29.0 (27.3-30.9)	<0.0001
Model 1	1 (reference)	1.10 (0.99-1.22)	1.09 (0.98-1.21)	1.49 (1.35-1.65)	<0.0001
Model 2	1 (reference)	1.09 (0.99-1.21)	1.07 (0.97-1.19)	1.45 (1.31-1.61)	<0.0001
Model 3	1 (reference)	1.05 (0.94-1.16)	1.02 (0.92-1.13)	1.34 (1.20-1.49)	<0.0001

Incidence rates are presented as rate/1,000 person years (95% confidence interval). Model data are presented as hazard ratio (95% confidence interval). For coronary heart disease (CHD), ischemic stroke, and total mortality, model 1 was adjusted by age, sex, race, total cholesterol,

HDL-C, SBP, antihypertensive medication, current smoking, and diabetes status (Pooled Cohort Equation variables); for heart failure (HF) hospitalization, model 1 was the ARIC heart failure model and included age, sex, race, SBP, antihypertensive medication, current smoking, diabetes status, BMI, and heart rate. Model 2 was model 1 plus eGFR. Model 3 was model 2 plus log NT-proBNP and log hs-TnT.

Table S3. Baseline characteristics across ARIC visit 5 galectin-3 quartiles (group 2; N=4829).

Risk factors	Quartiles of Galectin-3 (ng/mL)				P trend
	1st quartile (4.2-13.9) n=1218	2nd quartile (14.0-16.7) n=1211	3rd quartile (16.8-20.1) n=1195	4th quartile (20.2-104) n=1205	
Age (year)	74.3 ± 4.9	74.6 ± 4.8	75.9 ± 5.2	76.5 ± 5.4	<0.001
Female (%)	49.2	60.6	66.5	73.5	<0.001
Black (%)	16.9	20.5	24.4	29.1	<0.001
SBP (mmHg)	130 ± 19	130 ± 17	131 ± 18	131 ±19	0.38
Pulse pressure (mm Hg)	62 ± 15	62 ± 14	64 ± 14	65 ± 16	<0.001
Heart rate (beat/min)	64 ± 11	65 ± 11	66 ± 11	66 ± 11	<0.001
Hypertension (%)	62.1	69.6	70.3	83.5	<0.001
Current smoking (%)	6.0	5.5	6.2	5.4	0.710
Diabetes (%)	25.3	23.8	29.3	39.0	<0.001
BMI (kg/m^2)	27.5 ± 4.9	28.2 ± 5.4	28.3 ± 5.6	30.1 ±6.3	<0.001
Triglycerides (mg/dL)	102 (80, 139)	109 (84, 145)	114 (85, 148)	118 (92, 162)	<0.001
Total cholesterol (mg/dL)	187.8 ± 39.7	187.5 ± 40.3	188.5 ± 41.9	182.6 ± 41.7	0.001
HDL-C (mg/dL)	53.8 ± 14.3	53.6 ± 14.0	54.4 ± 14.8	51.6 ± 12.8	0.007
LDL-C (mg/dL)	110.6 ± 32.5	109.5 ± 33.8	109.3 ± 34.5	103.9 ±34.7	<0.001
eGFR (mL/min/1.73m^2)	77.8 ± 13.1	74.1 ± 14.4	70.5 ± 15.0	60.7 ± 18.3	<0.001
Chronic Kidney Disease (eGFR <60	10.3	17.7	25.0	48.6	<0.001

mL/min/1.73m^2) (%)					
NT-proBNP (pg/mL)	98.5 (52.2, 178.6)	97.4 (55.2, 194.8)	121.0 (65.9, 225.5)	163.5 (84.8, 316.6)	<0.001
hs-TnT (ng/L)	9 (6, 13)	9 (6,13)	10 (7, 14)	12 (8, 19)	<0.001
hs-TnI (ng/L)	2.6 (1.9, 4.1)	2.8 (1.9, 4.3)	2.9 (2.0, 4.5)	3.7 (2.4, 6.2)	<0.001
hs-CRP (mg/L)	1.61 (0.83, 3.41)	1.93 (0.91, 3.85)	2.03 (0.94, 4.22)	2.55 (1.24, 5.38)	<0.001
LVH (%)	3.0	4.5	5.3	6.4	<0.001

Data are presented as mean ± SD, median (25th, 75th percentiles), or percentage. P-values for linear trend were calculated by using trend test across ordered groups. SBP, systolic blood pressure, BMI, body mass index; eGFR, estimated glomerular filtration rate; HDL-C, high-density lipoprotein cholesterol; hs-TnT, high-sensitivity troponin T; hs-TnI, high-sensitivity troponin I; hs-CRP, high-sensitivity C-reactive protein; LDL-C, low-density lipoprotein cholesterol; LVH, left ventricular hypertrophy (Cornell ECG criteria); NT-proBNP, N-terminal pro–B-type natriuretic peptide.

Table S4. Cardiovascular outcomes by galectin-3 quartiles at ARIC visit 5 (group 2; N=4829).

	Galectin-3 quartiles (ng/mL)				P trend
	1st Quartile (4.2-13.9)	2nd Quartile (14.0-16.7)	3rd Quartile (16.8-20.1)	4th Quartile (20.2-104)	
**Incident total CHD**					
Incident rate	5.5 (3.8-7.8)	7.2 (5.3-9.9)	6.4 (4.5-9.0)	8.5 (6.3-11.5)	0.10
HR-model 1	*ref*	1.46 (0.89-2.39)	1.20 (0.70-2.03)	1.59 (0.95-2.67)	0.28
HR-model 2	*ref*	1.41 (0.86-2.33)	1.13 (0.66-1.93)	1.39 (0.80-2.41)	0.49
HR-model 3	*ref*	1.38 (0.84-2.27)	1.09 (0.64-1.86)	1.19 (0.68-2.08)	0.61
**Incident ischemic stroke**					
Incident rate	2.9 (1.78-4.7)	4.8 (3.3-7.0)	2.3 (1.3-4.1)	6.9 (5.0-9.6)	0.02
HR-model 1	*ref*	1.40 (0.74-2.64)	0.63 (0.29-1.38)	1.53 (0.81-2.92)	0.07
HR-model 2	*ref*	1.40 (0.74-2.66)	0.64 (0.29-1.40)	1.55 (0.78-3.08)	0.07
HR-model 3	*ref*	1.39 (0.73-2.63)	0.61 (0.28-1.35)	1.34 (0.67-2.69)	0.11
**Incident HF hospitalization**					
Incident rate	7.1 (5.2-9.7)	10.4 (8.0-13.5)	10.9 (8.4-14.1)	23.1 (19.2-27.7)	<0.0001
HR-model 1	*ref*	1.37 (0.88-2.13)	1.38 (0.89-2.14)	2.31 (1.53-3.50)	0.0002
HR-model 2	*ref*	1.32 (0.85-2.06)	1.28 (0.82-2.01)	1.96 (1.27-3.04)	0.012
HR-model 3	*ref*	1.28 (0.82-2.00)	1.15 (0.74-1.80)	1.43 (0.91-2.23)	0.41
**Death**					
Incident rate	14.6 (11.7-18.1)	17.2 (14.0-21.0)	21.6 (17.9-25.9)	33.1 (28.4-38.5)	<0.0001
HR-model 1	*ref*	1.12 (0.82-1.53)	1.21 (0.89-1.65)	1.79 (1.33-2.41)	0.0002
HR-model 2	*ref*	1.09 (0.80-1.50)	1.17 (0.85-1.59)	1.62 (1.19-2.22)	0.008
HR-model 3	*ref*	1.08 (0.79-1.49)	1.08 (0.79-1.48)	1.33 (0.97-1.84)	0.29

Incidence rates are presented as rate/1,000 person years (95% confidence interval). Model data are presented as hazard ratio (95% confidence interval). For coronary heart disease (CHD), stroke, and death, model 1 was adjusted by age, sex, race, total cholesterol, HDL-C, SBP,

antihypertensive medication, current smoking, and diabetes status; for heart failure (HF), model 1 was the ARIC heart failure model and included age, sex, race, SBP, antihypertensive medication, current smoking, diabetes status, BMI, and heart rate. Model 2 was model 1 plus eGFR. Model 3 was model 2 plus log NT-proBNP and log hs-TnT.

Table S5. Baseline characteristics at visit 4 across absolute change in galectin-3 levels from visit 4 to visit 5 (group 3; N=4981).

Risk factors	Δ Galectin-3 quartiles (ng/mL)				P trend
	**1st quartile (-32.4 to 1.1, n=1262)**	**2nd quartile (1.2-3.0, n=1273)**	**3rd quartile (3.001–5.4, n=1208)**	**4th quartile (5.401-69.5, n=1238)**	
Age (year)	60.0±4.9	60.5±4.9	61.3±5.1	62.1±5.4	<0.001
Female (%)	66.2	57.9	56.4	58.0	<0.001
Black (%)	17.7	17.4	23.3	24.6	<0.001
SBP (mmHg)	120 ± 16	122 ± 17	126 ± 17	130 ± 18	<0.001
Pulse pressure (mm Hg)	50 ± 13	51 ± 13	53 ± 13	58 ± 16	<0.001
Heart rate (beat/min)	65 ± 9	65 ± 9	65 ± 9	66 ± 9	0.052
Hypertension (%)	29.8	30.3	40.3	53.7	<0.001
Current smoking (%)	9.3	10.7	12.2	11.8	0.02
Diabetes (%)	5.2	6.3	12.0	19.4	<0.001
BMI (kg/m^2)	27.9 ± 5.1	27.9± 5.0	28.4 ± 5.0	29.7 ± 5.6	<0.001
Triglycerides (mg/dL)	119 (86, 166)	115 (84, 160)	120 (87, 168)	123 (91, 174)	0.007
Total cholesterol (mg/dL)	203.5 ± 35.7	200.7 ± 34.9	201.3±34.9	202.1±36.6	0.36
HDL-C (mg/dL)	52.9±16.4	52.3±17.2	51.0±16.0	49.8±16.1	<0.001
LDL-C (mg/dL)	123.4 ± 32.0	122.0 ± 32.1	122.8 ± 31.8	123.5 ± 33.7	0.93
eGFR (mL/min/1.73m^2)	88.5 ± 14.5	89.0 ± 13.4	88.9 ± 13.8	87.1 ± 15.6	0.006
Chronic Kidney Disease (eGFR <60	3.0	2.5	2.2	4.8	0.02

mL/min/1.73m^2) (%)					
NT-proBNP (pg/mL)	52.1 (27.2, 97.3)	50.7 (25.5, 97.9)	53.5 (25.8, 100.8)	64.9 (33.2, 121.7)	<0.001
hs-TnT (ng/L)	3 (1.5, 6)	4 (1.5, 6)	4 (1.5, 7)	5 (1.5, 8)	<0.001
hs-TnI (ng/L)	1.7 (1.2, 2.5)	1.9 (1.3, 2.6)	2.0 (1.3, 2.8)	2.1 (1.5, 3.1)	<0.001
hs-CRP	2.2 (1.0, 4.8)	1.8 (0.9, 4.3)	2.1 (1.0, 4.8)	2.6 (1.2, 5.1)	<0.001
LVH (%)	1.86	1.54	2.43	3.11	0.060

Data are presented as mean ± SD, median (25th percentile, 75th percentile), or percentage. P-values for linear trend were calculated by using trend test across ordered groups. SBP, systolic blood pressure, BMI, body mass index; eGFR, estimated glomerular filtration rate; HDL-C, high-density lipoprotein cholesterol; hs-TnT, high-sensitivity troponin T; hs-TnI, high-sensitivity troponin I; hs-CRP, high-sensitivity C-reactive protein; LDL-C, low-density lipoprotein cholesterol; LVH, left ventricular hypertrophy (Cornell ECG criteria); NT-proBNP, N-terminal pro–B-type natriuretic peptide.

Figure S1. Age-, race-, and sex-adjusted survival curves assessing the time to incident coronary heart disease (CHD), ischemic stroke, heart failure hospitalization (HF), and total mortality across galectin-3 quartiles at visit 4.

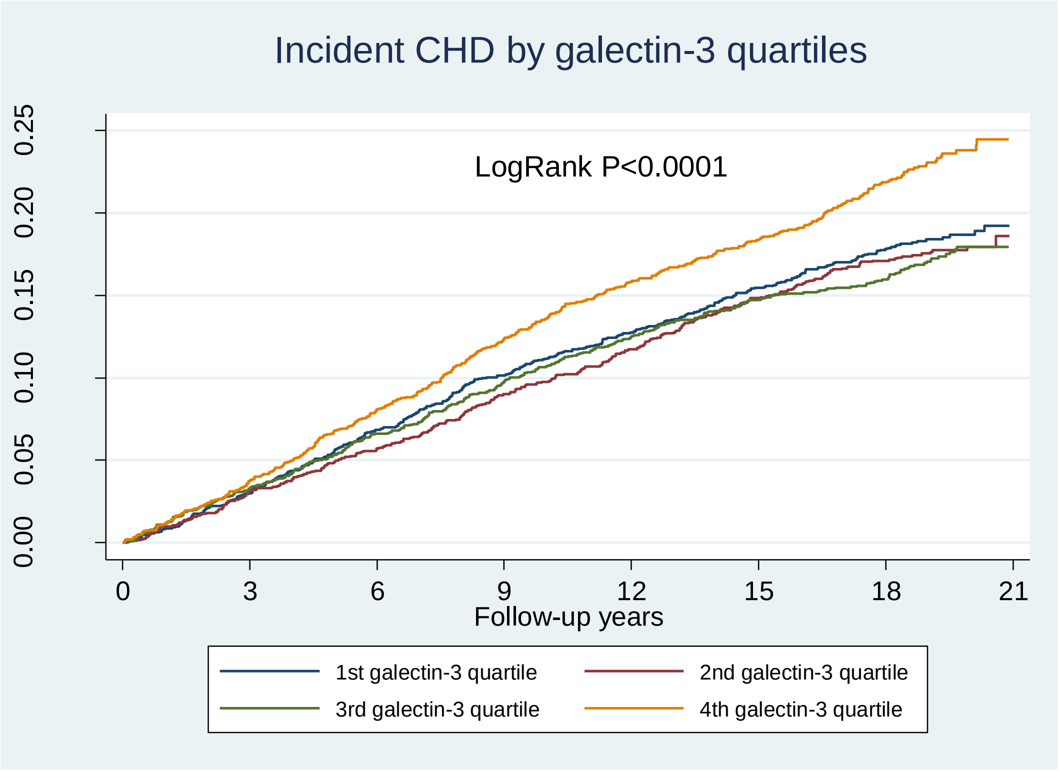

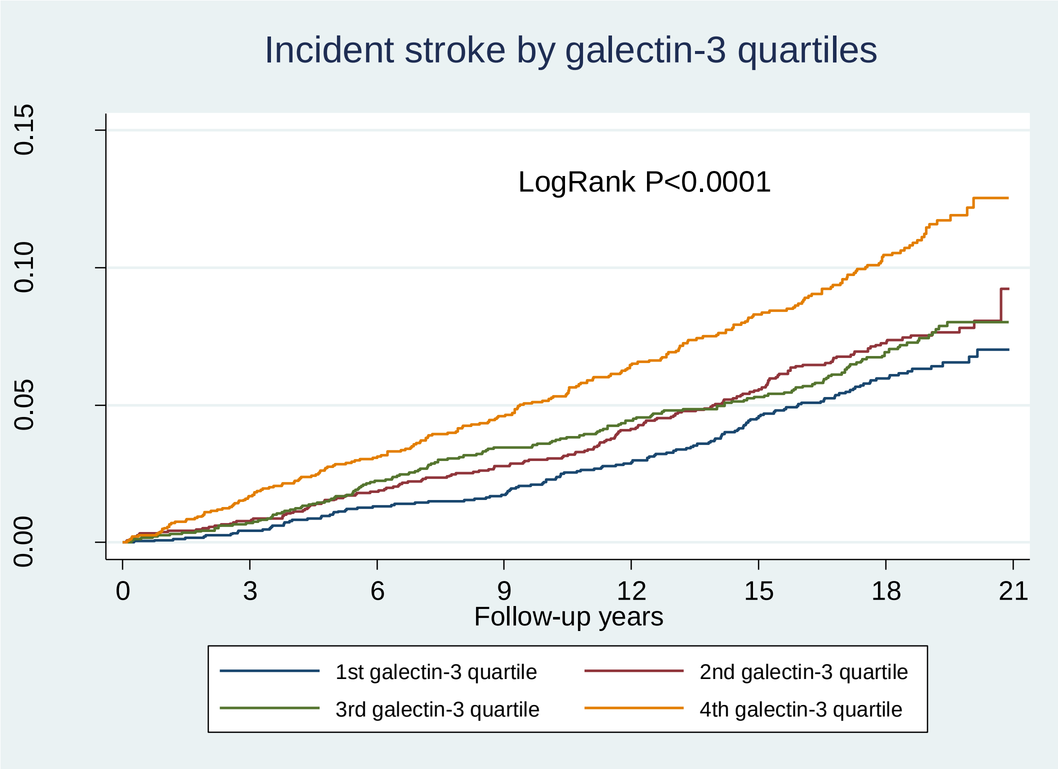
Incident stroke by galectin-3 quartiles
LogRank P<0.0001
0.00
0.05
0.10
0.15
0
3
6
9
12
15
18
21
Follow-up years
1st galectin-3 quartile
2nd galectin-3 quartile
3rd galectin-3 quartile
4th galectin-3 quartile

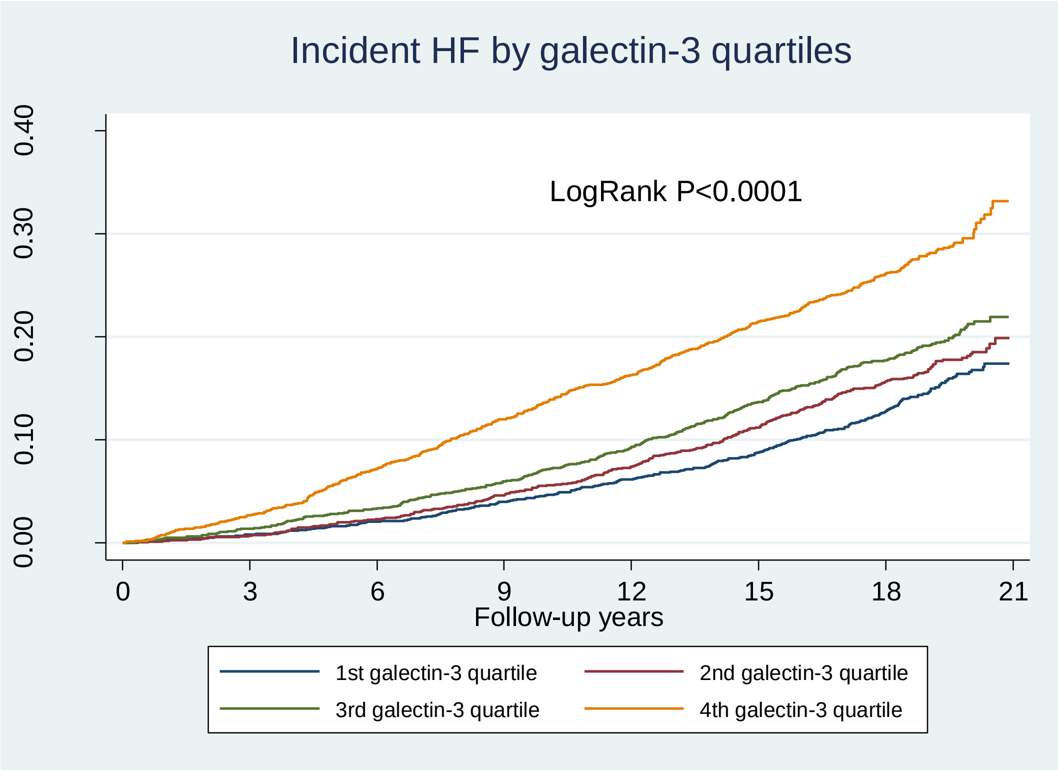
Incident HF by galectin-3 quartiles
LogRank P<0.0001
0.00
0.10
0.20
0.30
0.40
0
3
6
9
12
15
18
21
Follow-up years
1st galectin-3 quartile
2nd galectin-3 quartile
3rd galectin-3 quartile
4th galectin-3 quartile

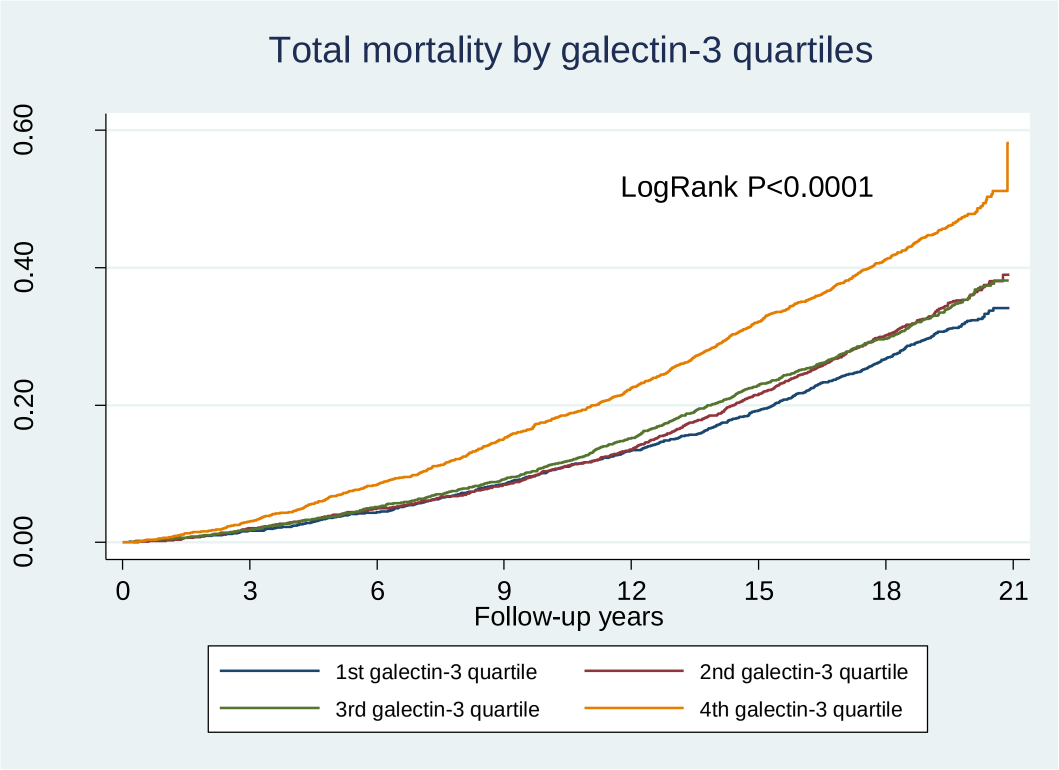

P-values were calculated by log-rank test for equality of survival function. Crude incidence rates were calculated as number of events per 1000 person-years.
